# The VHL/HIF Axis in the Development and Treatment of Pheochromocytoma/Paraganglioma

**DOI:** 10.3389/fendo.2020.586857

**Published:** 2020-11-24

**Authors:** Song Peng, Jun Zhang, Xintao Tan, Yiqiang Huang, Jing Xu, Natalie Silk, Dianzheng Zhang, Qiuli Liu, Jun Jiang

**Affiliations:** ^1^ Department of Urology, Daping Hospital, Army Medical University, Chongqing, China; ^2^ Department of Bio-Medical Sciences, Philadelphia College of Osteopathic Medicine, Philadelphia, PA, United States

**Keywords:** pheochromocytomas, paragangliomas, VHL, HIF, metabolism, inhibitor

## Abstract

Pheochromocytomas and paragangliomas (PPGLs) are rare neuroendocrine tumors originating from chromaffin cells in the adrenal medulla (PCCs) or extra-adrenal sympathetic or parasympathetic paraganglia (PGLs). About 40% of PPGLs result from germline mutations and therefore they are highly inheritable. Although dysfunction of any one of a panel of more than 20 genes can lead to PPGLs, mutations in genes involved in the VHL/HIF axis including *PHD*, *VHL*, *HIF-2A (EPAS1)*, and *SDHx* are more frequently found in PPGLs. Multiple lines of evidence indicate that pseudohypoxia plays a crucial role in the tumorigenesis of PPGLs, and therefore PPGLs are also known as metabolic diseases. However, the interplay between VHL/HIF-mediated pseudohypoxia and metabolic disorder in PPGLs cells is not well-defined. In this review, we will first discuss the VHL/HIF axis and genetic alterations in this axis. Then, we will dissect the underlying mechanisms in VHL/HIF axis-driven PPGL pathogenesis, with special attention paid to the interplay between the VHL/HIF axis and cancer cell metabolism. Finally, we will summarize the currently available compounds/drugs targeting this axis which could be potentially used as PPGLs treatment, as well as their underlying pharmacological mechanisms. The overall goal of this review is to better understand the role of VHL/HIF axis in PPGLs development, to establish more accurate tools in PPGLs diagnosis, and to pave the road toward efficacious therapeutics against metastatic PPGLs.

## Introduction

Pheochromocytomas (PCCs) are catecholamine-secreting tumors that originated from the chromaffin cells in the adrenal medulla. Paragangliomas (PGLs) are neural crest-derived neuroendocrine neoplasms originating from extra-adrenal sympathetic or parasympathetic ganglia (1). Both PCCs and PGLs are collectively known as PPGLs. PPGLs are rare tumors with the incidence rate between 0.2 and 0.8 per 100,000 (2–4) with great clinical manifestations (5). Due to elevated levels of catecholamines in the circulation, the common clinical presentations of PPGLs include episodes of headache, sweating, palpitation, and hypertension. In addition, about 10% of PCCs are metastatic (6) and 40% of PGLs are considered as metastatic disease (7, 8).

Etiologically, about 70%–80% of PPGLs are caused by genetic abnormalities which affect different signaling pathways (9). Approximately, 40% of PPGLs result from germline mutations, and therefore they are highly inheritable (10). Although dysfunction of any of these related susceptible gene products can lead to PPGLs, mutations in the genes encoding the VHL/HIF axis such as *VHL*, *HIF*, and *PHD* are more commonly found in PPGLs (11). Moreover, multiple lines of evidence suggest that pseudohypoxia plays a crucial role in the tumorigenesis of PPGLs. In this review, we will discuss the genetic alterations affecting the VHL/HIF axis and dissect the underlying molecular mechanisms in pseudohypoxia signaling and PPGLs. We will also summarize the currently available compounds or drugs targeting VHL/HIF axis, their specific targets, and pharmacological mechanisms.

## The VHL/HIF Axis

**Figure 1:**
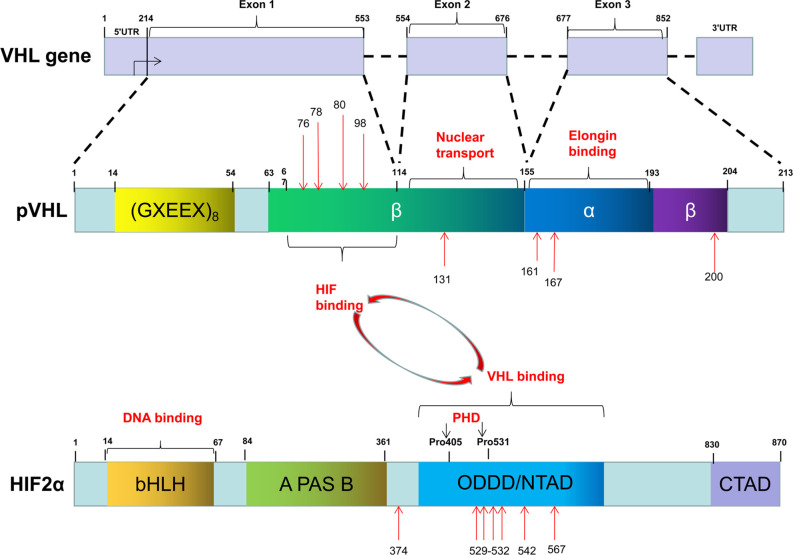
The common mutation sites of VHL and EPAS1 genes in PPGLs.

The Von Hippel-Lindau (*VHL*) gene located on 3p25.5 encodes an ancient tumor suppressor, pVHL. Although pVHL functions in both physiology and pathology, as a component of an E3 ubiquitin-ligase complex, pVHL plays a determinant role in the degradation of hypoxia-induced factors (HIFs) including HIF-1α, HIF-2α, and HIF-3α. The roles of HIF-1α and HIF-2α in sensing and facilitating cellular adaptation to hypoxic conditions as well as their underlying molecular mechanisms are well-established (12). However, much less is known about HIF-3α. Functionally, HIF-1α and HIF-2α heterodimerize with HIF-β by HLH domain, which is also known as ARNT, to transcriptionally regulate a wide spectrum of HIF target genes. Both HIF-α and HIF-β belong to the basic helix-loop-helix-Per-ARNT-Sim (bHLH-PAS) family. They contain a basic DNA binding domain, a conserved NH_2_-terminal domain (N-TAD), and two specialized transactivation domains located in their variable COOH-terminal domains (C-TAD) (13) (**Figure 1**). The asparagine residue (N803) in the C-TAD of HIF-α can be hydroxylated by factor-inhibiting HIF (FIH) to interrupt its interaction with CREB-binding protein (CBP)/p300, an essential coactivator of HIF (14–16). The N-TAD also contains an oxygen-dependent domain (ODDD), in which a few prolyl residues (Pro-402 and Pro-564 in HIF-1α; Pro-405 and Pro-531 in HIF-2α) are selectively hydroxylated under normoxic condition and hydroxylated HIFs are subsequently degraded (17–20). The enzymes responsible for HIF-α hydroxylation belong to the egg-laying-defective nine (*EGLN*) family known as PHD1, PHD2, and PHD3 because they all contain a prolyl-4-hydroxylase domain. These enzymes are dioxygenases and use both molecular oxygen and Fe^2+^ as their co-substrates to catalyze HIF-α hydroxylation.

**Figure 2:**
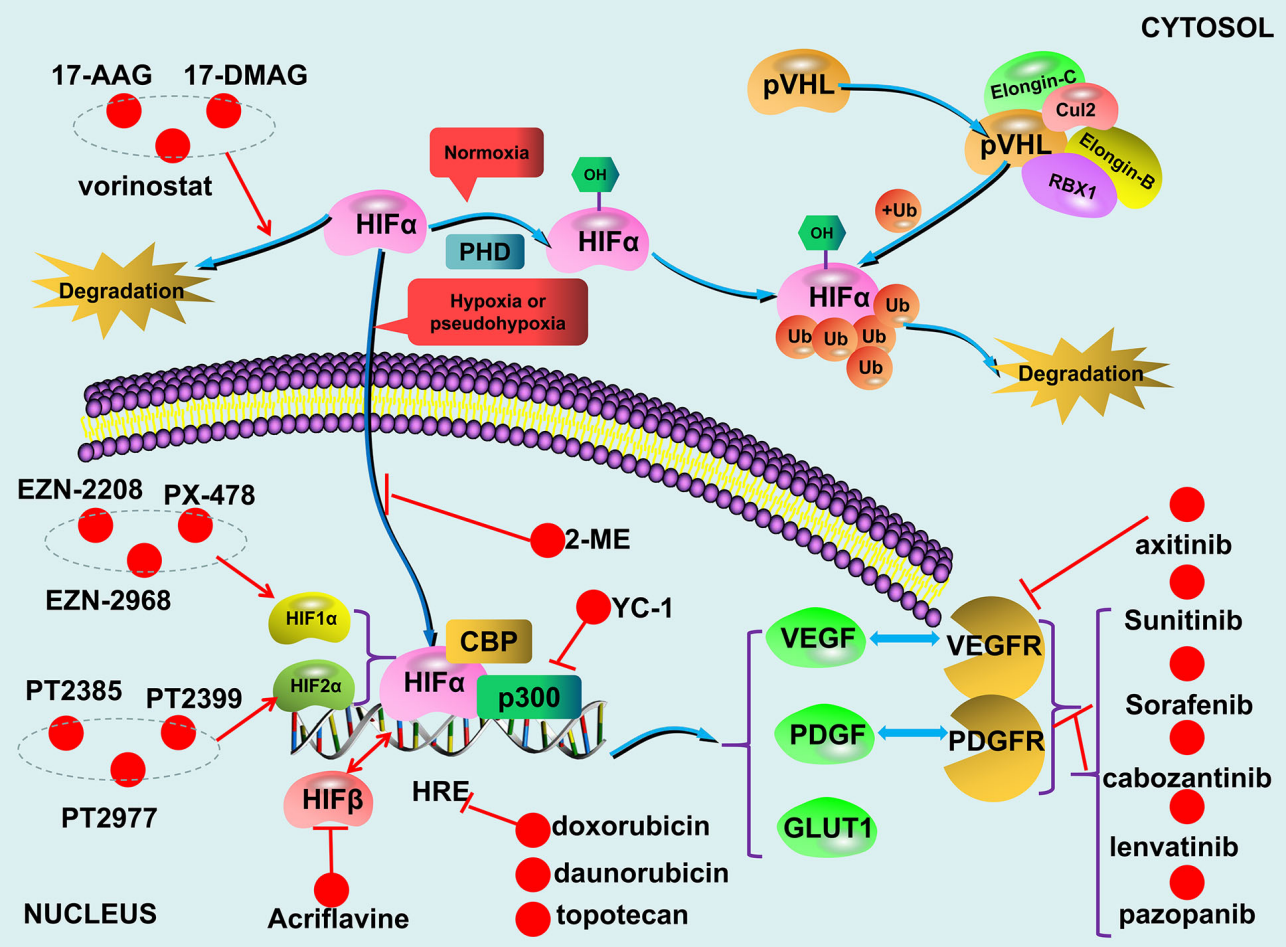
The VHL/HIF axis and compounds targeting the axis.

The VHL/HIF axis responses to reduced oxygen concentration or hypoxia. Although HIF-1α and HIF-2α have about 48% sequence similarity, they regulate two different groups of target genes with limited overlap mainly due to their dissimilar transactivation domains (21, 22). In addition, HIF-1α is widely expressed, while HIF-2α is only expressed in certain cell types (23, 24). For example, the genes involved in glucose metabolism are mainly regulated by HIF-1α. HIF-2α plays a more important role in the adjustment to high altitudes and the regulation of EPO expression (25, 26). As mentioned above that compared to HIF-1α and HIF-2α, much less is known about HIF-3α. Since it lacks the transactivation domain (27), HIF-3α likely does not transcriptionally regulate its target genes. Overall, the levels and functions of both HIF-1α and HIF-2α are oxygen-concentration dependent. Specific proline residues of HIF-1α and HIF-2α are hydroxylated by PHD under normoxic conditions. With the involvement of the molecules such as elongin B, elongin C, cul2, the hydroxylated HIFs are recognized by the pVHL (28–30), subsequently ubiquitinated and ultimately degraded (31, 32). Under hypoxic conditions, the non-hydroxylated HIFs are dissociated from pVHL, accumulated in the cells, and subsequently upregulate their target genes transcriptionally. However, failure in the degradation of HIFs due to either deletion or mutation of either *VHL*, *HIFs*, or *PHDs* can lead to dysregulation of HIFs-regulated genes in a variety of diseases including PPGLs (**Figure 2**).

## Dysregulation of the VHL/HIF Axis and PPGLs

As mentioned above that mutation in either the three genes encoding pVHL, HIFs and PHDs can lead to abnormal accumulation of HIFs. Minor alteration of this axis usually causes erythrocytosis; whereas major dysregulation of the axis is associated with tumorigenesis (33). Although a wide spectrum of tumors including hemangioblastomas, renal cell carcinoma (RCC), pancreatic neuroendocrine tumor, and PPGLs can result from dysregulation of the VHL/HIF axis (34–37), this review will only focus on the relationship between aberrations of these genes and PPGLs.

### VHL Mutations

After the *VHL* mutations were first described in an ophthalmic disease (34), multiple studies subsequently confirmed that *VHL* mutations can cause a variety of diseases including cancers (35–37). To honor the contributions of the German ophthalmologist Eugen von Hippel and the Swedish pathologist Arvid Lindau, the gene responsible for these diseases is, therefore, named as *VHL*. Of note, VHL disease caused by heterozygous germline mutations is autosomal dominant and almost completely penetrant (97%) (38). VHL diseases are generally classified into two types, type 1 (without PCCs) and type 2 (with PCCs). The type 2 disease is manifested as RCCs, PCCs, central nervous system, retinal hemangioblastomas, pancreatic neuroendocrine tumors and pancreatic and renal cysts and can be further divided into three subtypes (34), PCCs with all types of VHL disease manifestations without RCC (Type 2A), PCC with all types of VHL disease including RCC (Type 2B), and isolated PCCs (Type 2C).

To date, more than 1,000 mutations in *VHL* gene have been identified. These mutations can be categorized as missense mutation (52%), frameshift mutation (13%), nonsense mutation (11%), in-frame deletion/insertion mutation (6%), large/complete deletion mutation (11%), and splicing mutation (7%) (39). The common germline mutations in *VHL* are delPhe76, Asn78Ser, Argl61Stop, Arg167Gln, Argl67Trp, and Leu178Pro (40) (**Figure 1**). Recently, we reported four missense mutations in five Chinese unrelated families c.239G>T (p.Ser80Ile), c.232A>T (p.Asn78Tyr), c.500G>A (p.Arg167Gln), c.293A>G (p.Try98Cys), and all four mutations predispose the patients to VHL disease (41). Notably, type 2 VHL disease mainly resulted from missense mutations (85%–92%) (40, 42), especially mutations in codons 167 and 238, are mainly associated with PPGLs (43, 44). In contrast, homozygous germline mutations are rare or barely cause tumors. Sonny et al. found a c.598C>T (p.Arg200Trp) homozygous missense germline mutation of *VHL* caused Chuvash polycythemia (45). In addition, somatic *VHL* mutations were found in majority (50%–70%) of clear-cell RCC cases (38).

It has been reported that different mutations in *VHL* lead to diverse clinical symptoms (41, 46–49), and sometimes even the same mutation can lead to different phenotypes (50–53). Since pVHL has multiple functional domains, one of the potential explanations for this phenomenon is that a specific mutation causes particular dysfunction. It appears that missense mutations are more likely linked with type 2 disease and truncating mutations are responsible for type 1 disease (54). However, Liu et al. further stratified the missense mutations as HIF-α binding site missense mutations (HM) group and non-HIF-α binding site missense mutations (nHM) group, and found that the missense mutations in HM group had similar risks of most tumors with truncating mutations with the exception that the HM group had a lower risk of RCC. Moreover, compared to nHM, missense mutations in HM had a higher risk of pancreatic cyst or tumor and a lower risk of PCCs (55). Secondly, some functions of pVHL are O_2_-independent (56, 57) or unrelated to HIF regulation, these functions may also be involved in PPGLs pathogenesis. Michael et al. found that RCCs with deficient pVHL exhibited deficiency in fibronectin matrix assembly (58). Intriguingly, Clifford et al. reported that mutations associated with type 2C phenotype could even promote, rather than inhibit, HIF-α ubiquitylation and degradation (39). These findings altogether supported the notion that disturbing the functions of pVHL contributes to the development of PPGLs. Additionally, based on Knudson’s Two-Hit model (59), it is understandable that the diverse phenotypes of VHL diseases could be the result of two different “hits”.

The VHL/HIF axis also can be affected by dysregulated epigenetic modifications such as gene silencing by methylation of the CpG islands in the promoter of related genes. Indeed, promoter hypermethylation occurs in about 3%–42% of clear-cell RCC (60). Adam Andreasson found that the promoter methylation of the *VHL* gene is not only elevated in PPGLs compared with normal tissue (57% vs. 27%) but also significantly higher in malignancies than that in tumors (63% vs. 55%) (61). However, the precise molecular mechanisms in the pathogenesis of PPGLs related to loss-of-function of pVHL are still largely unknown and therefore need further investigation.

### HIF-A Mutations

As mentioned above that HIF-α family composed three members, HIF-1α, HIF-2α, and HIF-3α. But little is known about HIF-3α. Compared with *HIF-2A*, *HIF-1A* has relatively few mutations, ClinVar database (https://www.ncbi.nlm.nih.gov/clinvar/) only collects 30 records. Morris et al. reported a somatic mutation (p.Val116Glu) and a germline missense mutation (p.Ala475Ser) of *HIF-1A* in a clear-cell RCC with *VHL* inactivation. Of note, the germline mutation (p.Ala475Ser) was likely to be a benign variant (62). Furthermore, Gladek et al. found that *HIF-1A* Single-Nucleotide Polymorphisms (SNPs) are association with the phenotypes of many tumors (63). In PPGLs patients, only copy number aberration (TCGA-QT-A5XP, https://portal.gdc.cancer.gov/), not *HIF-1A* mutation, have been found. On the other hand, both germline and somatic mutation in *HIF-2A* have been identified in patients with polycythemia and/or PPGLs. However, it appears that germline mutations of *HIF-2A* including p.Met535Val, p.Gly537Arg, p.Gly537Trp only leads to polycythemias, not tumors (64, 65). A gain-of-function germline mutation in *HIF-2A* alone is not sufficient for tumorigenesis presumably that simultaneous loss-of-function in some tumor suppressors is needed. In fact, we recently reported that germline mutations in *HIF-2A* (c.1609G>A, p.Gly537Arg) are responsible for polycythemia formation and additional somatic *VHL* mutations are needed for the development of clear-cell RCC (66). Similarly, a germline mutation in *HIF-2A* exon 9 (c.1121T>A, p.F374Y) leads to polycythemia and predisposes the patients for PPGLs development (67). In addition, somatic mutations in *HIF-2A* appear to be more frequent genetic events in PPGLs (68). For example, Zhang et al. reported two gain-of-function somatic mutations (c.1588G>A, p.Ala530Thr and c.1589C>T, p.Ala530Val) in exon 12 of *HIF-2A* resulting in paraganglioma and polycythemia, respectively. Further analyses suggest that mutations in the vicinity of the hydroxylation site Pro-531 affect the catalytic activity of PHD and then lead to the interrupted interaction between HIF-2α and pVHL (69). Moreover, Karel Pacak et al. reported two somatic mutations of *HIF-2A* (c.1595A>G p.Y532C and c.1586T>C p.L529P) in patients with either congenital polycythemia, multiple recurrent PPGLs, or somatostatinoma (70). We recently found that a gain-of-function mutation of *HIF-2A* (c.1589C>T) leads to PPGLs with polycythemia simultaneously (26) and a mutation in *HIF-2A* immediately distal to its DNA binding domain (p.Ser71Tyr) has been identified in sporadic PPGLs (71) (**Figure 1**). Germline or somatic mutations of *HIF-2A* can be mosaic. Buffet et al. reported two cases of *HIF-2A*-related Polycythemia-Paraganglioma Syndrome resulted from mosaicism mutations. They found that these patients could present with young age and multiplicity; and also the mutations could be transmitted to the offspring (72). In addition, *HIF-2A* mosaic mutation might be involved in high secretion of catecholamines and cyanotic congenital heart disease (73).

### Mutation in PHD and Other Related Factors

Heterozygous germline mutations in *PHD2* gene were first reported in familial erythrocytosis (74, 75). Later, Ladroue et al. reported a heterozygous loss-of-function mutation of *PHD2* (c.1121A>G, p.His374Arg) with the development of both erythrocytosis and recurrent paraganglioma. Functional analysis indicates that His374 is important in the binding of cofactor Fe2+, and mutation of this residue is expected to impair the catalytic function of PHDs (76). Yang et al. reported heterozygous germline mutations in *PHD1* (c.188T>A, p.Ser61Arg and c.682G>T, p.Ala228Ser) in patients with polycythemia and PPGLs, respectively. Further research found that the half-lives of both PHD1 and PHD2 are reduced with these *PHD1* mutants (77). These findings collectively demonstrated that mutant *PHDs* are indeed associated with susceptibility to PPGLs. However, compared to *VHL* and *HIF-A*, mutations in *PHDs* are relatively rare in patients with PPGLs (78). Additionally, mutations of enzymes in the TCA cycle can affect VHL/HIF axis indirectly. For example, elevated levels of HIFs can be caused by the mutations in *SDHx*, *FH*, *MDH*, and *IDH* with subsequent accumulation of specific metabolites and reactive oxygen species (31, 79–89). In addition, multiple lines of evidence indicated that mutations in cluster 2 (Kinase Signaling Cluster) genes, including *NF1*, *RET*, *TMEM127*, *ERK*, *MAX*, and *H-RAS* could affect the VHL/HIF axis indirectly (90–94), although these mutations were initially thought to drive PPGLs through the oxygen-independent kinase signaling pathway, such as mTOR axis.

## The Mechanisms in Dysregulated VHL/HIF Axis and PPGLs

Under normal physiological conditions, HIFs are degraded during normoxic condition and HIFs accumulation only occur during hypoxia. The undegraded HIF-α translocates to the nucleus and dimerizes with HIF-β (95). Together with p300/CBP, the HIF-α/HIF-β heterodimer is recruited to the hypoxia-responsive elements (HREs) located on the promoter regions of HIF-regulated targets to transcriptionally upregulate the expression of the genes including vascular endothelial growth factor (*VEGF*), platelet-derived growth factor (*PDGF*), and glucose transporter (*GLUT*) (93, 96–98) (**Figure 2**). The combined effects of these upregulated gene products result in an increased supply of blood and nutrients to the hypoxic tissues and switch glucose metabolism from aerobic to anaerobic glycolysis. Due to the fast growth of tumor tissues, this process occurs in all solid tumors (99, 100), and dysregulated VHL/HIF axis further exacerbate the development of certain tumors such as PPGLs.

Aerobic glycolysis, also known as Warburg Effect (9, 101, 102), occurs in all solid tumor cells. However, dysregulated VHL/HIF axis plays a more important role in certain cancer types such as clear-cell RCC and PPGLs. Pseudohypoxia, mimicking the hypoxic condition, can affect different cancer processes including tumorigenesis and malignant transformation by promoting epithelial-mesenchymal transition and enhancing stem cell-like property. Of note, metabolic reprogramming can affect each of these processes and the role of VHL/HIF axis in cancer metabolic reprogramming has been well defined. HIF-1 aberrant activation due to either *VHL* or *PHD* mutations increases glucose uptake and glycolysis with a concomitant decrease in mitochondrial mass (103). HIF-α, especially HIF-1α, controls a wide spectrum of enzymes including GLUT1, GLUT3, hexokinase 1/2, lactate dehydrogenase-A (LDH-A), and pyruvate dehydrogenase kinase 1 (PDK1) (104–108). Upregulating these enzymes collectively shifts glycolysis from aerobic to anaerobic (109).

PPGLs are also considered as metabolic diseases due to the increased secretion of one or more catecholamines (epinephrine, norepinephrine, and dopamine). Catecholamines play a crucial role in the regulation of multiple metabolic pathways. Patients with PPGLs usually manifest with impaired insulin secretion, increased insulin resistance, elevated lipolysis, and the bone resorption marker C-terminal telopeptide of type I collagen (110). Many studies have revealed that oncometabolite such as succinate, fumarate, and 2-hydroxyglutarate (2HG) are increased in PPGLs (83, 111, 112). Another study found that compared to PPGLs without *SDHx* mutation, PPGLs with a deficient SDH have 25-fold higher succinate and 80% lower levels of fumarate, cis-aconitate, and isocitrate (113). Mutation in *FH* and *IDH* lead to the accumulation of fumarate and (R)-2-hydroxyglutarate, respectively (88, 114). Mechanistically, these oncometabolite modulate the activity of α-ketoglutarate-dependent dioxygenases such as PDH, which are involved in the induction of the pseudohypoxia pathway and activation of HIF axis (10, 31, 115). In addition, PPGLs with a germline mutation in genes encoding enzymes in the TCA cycle belong to Cluster I tumors, characterized by a pseudohypoxia signature (31). Together with the other intermediate metabolites of the TCA cycle, succinate can increase the chance of tumor development and progression through an ill-defined mechanism (83).

Results from more recent researches indicate that HIFs can regulate non-coding RNA (ncRNA) either directly or indirectly. Direct regulation is achieved by the recruiting HIFs to the HREs located on the promoter regions of ncRNAs. Whereas indirect regulation of ncRNA is achieved by epigenetic modification (116). One of the HIFs targets microRNA 210 (miR-210) (117) participates in a variety of biological processes including carcinogenesis, cancer cell proliferation, apoptosis, angiogenesis, and metastasis (118–120). On the other hand, miRNA can also activate HIF *via* mTOR indirectly. Calsina et al. reported miR-21-3p can regulate TSC2/mTOR axis in metastatic PPGLs and proposed that miR-21-3p can be the predictive markers of metastases (121). In addition, some lncRNA such as H19, MALAT1, HOTAIR, and lncRNA-SARCC play important roles in the activity of VHL/HIF axis (122).

## Inhibitors Targeting The VHL/HIF Axis

Since the VHL/HIF axis plays a critical role in the development of PPGLs, targeting this axis could be a promising therapeutic strategy. Multiple reagents targeting the VHL/HIF axis have been explored and some of them have been applied clinically (123–127). Among them, the tyrosine kinase inhibitors (TKIs) are most widely used because TKIs can repress angiogenesis by inhibiting the VEGF pathway (128–130). Some compounds targeting the VHL/HIF axis can inhibit tumor growth in both animal models and clinical trials (**Table 1**).

**Table 1:** The inhibitors targeting the VHL/HIF axis.

	Drugs or compounds	Targets or mechanisms	Clinical trials for PPGLs (www.clinicaltrials.gov)
Tyrosine kinase inhibitors	Sunitinib	Targeting VEGFR-1,2, PDGFR-β,RET, FGFR	NCT01371201, NCT00843037
	Sorafenib	Targeting RAF kinase, c-KIT, FLT-3, RET, VEGFR1-3, and PDGFR-β	None
	Cabozantinib	Targeting VEGFR, MET, RET	NCT02592356, NCT04400474, NCT02302833
	Axitinib	Targeting VEGFR	NCT01967576, NCT03839498
	Lenvatinib	Targeting VEGFR, FGFR, RET, c-Kit, PDGFα	NCT03008369, NCT02592356
	Pazopanib	Targeting VEGFR1-3, PDGFR-α,β, c-Kit	NCT01340794
Non-selective HIFs inhibitors	17-AAG	Promoting protein degradation	None
	17-DMAG	Promoting protein degradation	None
	Vorinostat	Promoting protein degradation	None
	Topotecan	Inhibiting translation and transcription activity	None
	Acriflavine	Inhibiting heterodimerization	None
	2-Methoxyestradiol	Inhibiting nuclear translocation and transcriptional activity	None
	YC-1	Inhibiting protein accumulation and transcription activity	None
	Doxorubicin/daunorubicin	Inhibiting DNA binding	NCT00002764, NCT00002608, NCT00002641
HIF-1α inhibitors	PX-478	Inhibiting mRNA expression and translation	None
	EZN-2208	Inhibiting mRNA expression	None
	Chetomin	Disrupting binding to p300	None
	Echinomycin	Inhibiting DNA binding	None
	KC7F2	Inhibiting protein synthesis	None
	Glyceollins	Inhibiting protein synthesis and stability	None
	Bisphenol A	Promoting protein degradation	None
	LW6	Promoting protein degradation	None
	PX-12	Promoting protein degradation	None
	Cryptotanshinone	Blocking nuclear translocation	None
	cyclo-CLLFVY	Inhibiting heterodimerization	None
	Indenopyrazole 21	Inhibiting transcriptional activity	None
	EZN-2968	Inhibiting mRNA expression and translation	None
HIF-2α inhibitors	PT2385	Inhibiting heterodimerization	None
	PT2399	Inhibiting heterodimerization	None
	PT2977	Inhibiting heterodimerization	None

### Tyrosine Kinase Inhibitors

To date, more than 40 protein kinase inhibitors have been approved by the FDA for cancer treatment (131). Several TKIs including sunitinib, cabozantinib, axitinib, Lenvatinib, and pazopanib are currently being evaluated in phase II clinical trials (www.ClinicalTrials.gov). By repressing the tyrosine kinase receptors, these reagents can inhibit cancer cell growth, metastasis, and the development of therapeutic resistance (132). More recently, several case studies and/or clinical trials in small cohorts suggest that TKIs could be a promising treatment for metastatic PPGLs or the syndrome-associated PPGLs.

Sunitinib, an orally administered TKI, can target both VEGFR and PDGFR (133), and therefore, it could potentially serve as a therapeutic reagent for PPGLs treatment. Early *in vitro* studies showed that sunitinib can repress the growth of PCCs (134), inhibit both synthesis and secretion of catecholamine (135). Several clinical trials have suggested that patients with metastatic PPGLs responded well to sunitinib (136–140). Results from one of our recent studies also suggested that sunitinib could be an optional therapy for patients with VHL disease-associated PCCs (141). Results from the SNIPP trial showed that sunitinib at 50mg daily benefited most patients with progressive PPGLs. Of 23 evaluable cases, the disease control rate (DCR) was 83% and median progression free survival (PFS) was 13.4 months, 3 (13%) patients with germline variants in *RET* or *SDHx* achieved a partial response (PR), 16 (70%) patients had stable disease (SD) (142). Currently, a phase II clinical trial (the First International Randomized Study in Malignant Progressive Pheochromocytoma and Paraganglioma, FIRSTMAPP) studying the effect of sunitinib on PPGLs is ongoing. In addition, results from sdhb knockout tumors bearing mice showed that sunitinib treatment can prevent tumor growth and vessel development in the first 2 weeks; thereafter, resistance will develop (143). Another study by using both *in vivo* and vitro models demonstrated that sunitinib and sorafenib can inhibit the growth of PCCs (144, 145). Previous study reported that a patient with recurrence and metastatic PPGLs responded well to 12 weeks of sorafenib treatment evidenced by regressed metastatic and decreased catecholamine level (146).

In addition, cabozantinib also appears to be a promising TKI for patients with PPGLs, especially for those with bone metastases. A trial (NCT02302833) enrolled 11 PPGLs patients with bone metastases is currently ongoing. Preliminary results identified 4 patients with PR (37%) and 6 patients with SD (55%); all patients with SD had tumor regression (18%–29%). The DCR was 92%, PFS was 16 months. None of the patients had any serious hypertension or cardiovascular events (147). A recent trial (NCT01967576) showed that 36% of patients with metastatic PPGLs achieved a PR when treated with axitinib (148); while only one of seven patients with metastatic PPGLs who received pazopanib showed a PR (149). Finally, recruitment for a phase II clinical trial has just begun to test if lenvatinib can be used as an anti-angiogenic medication for metastatic PPGLs (www.ClinicalTrials.gov) (**Figure 2**).

Although the promising therapeutic effects of TKIs on PPGLs have been widely reported, the toxicity of TKIs should also be mentioned. The side effects of TKIs include fatigue, nausea, thrombocytopenia, hypertension, myocardial infarction, and restrictive cardiomyopathy and so on. O’Kane et al. reported that due to severe adverse events, several patients needed to reduce the dose of sunitinib, and even 20% patients discontinued trial participation (142). A phase III clinical trial compared the safety of pazopanib and sunitinib in metastatic RCC, the results showed that patients treated with sunitinib had a higher incidence of fatigue, the hand-foot syndrome and thrombocytopenia than patients treated with pazopanib. Although the rate of cardiovascular adverse events of pazopanib were similar to that of sunitinib, the abnormal liver tests leading to discontinuation in pazopanib-treatment patients should be noted (139). Furthermore, the tolerance of axitinib was similar to that of other VEGFR inhibitors. Rini et al. reported that axtinib more frequently causes hypertension than sorafenib (40% vs. 29%) (NCT00678392) (140). Similarly, Van Geel et al. reported that the incidence of hypertension in axtinib-treatment patients was higher than that in pazopanib-treatment patients (150). Burotto Pichun et al. reported that even 80% axtinib-treatment patients developed severe hypertension (148). Recently, a phase III randomized ATLAS trial assessed the safety of axitinib versus placebo, axitinib-treated patients had more grade 3/4 adverse events and discontinuations (151). Taken together, the safety of TKIs needs to be further evaluated in the future.

### HIFs Inhibitors

Transcription factors including HIFs have been historically considered undruggable. This is one of the reasons that research in the pharmaceutical field has been mainly focusing on HIF’s downstream pathways, such as VEGF. However, based on the structure of HIF-2α (152), two compounds PT2385 and PT2399 targeting HIF-2α were successfully identified (145, 153). Subsequent *in vitro* and *in vivo* studies showed that these compounds can inhibit the growth of clear-cell RCC (154). A phase I trial found that for patients with progressive clear-cell RCC the complete response, partial response, and stabilized disease to PT2385 were 2%, 12%, and 52%, respectively (155). It has been proposed that HIF-2α inhibitors possess a great potential for the treatment of advanced PPGLs (156). These initial results could also spearhead a multitude of preclinical and clinical studies assessing the efficiency of the compounds in other tumor types. In fact, PT2385 has entered its phase II clinical trial (NCT03108066) evaluating its efficacy in patients with advanced cancers carrying a *VHL* germline mutation. Recently, second-generation allosteric inhibitor of HIF-2α PT2977 (MK-6482) was identified. Compared to PT2385, PT2977 have increased potency and improved pharmacokinetic profile (157). The result of phase I/II trial of PT2977 in 55 patients with advanced RCCs revealed that 24% patients experienced a confirmed PR and 54% had SD, with a clinical benefit rate of 78%. Moreover, a PT2977 monotherapy Phase III trial in patients with previously treated advanced RCC is planned (158). Notably, previous studies reported that HIF-2α was overexpressed in VHL and in SDH-related PPGLs compared to HIF-1α (159, 160). Therefore, inhibitors targeting HIF-2α appear to be more promising than inhibitors targeting HIF-1α.

### Other Compounds Targeting VHL/HIF Axis

Theoretically, any compounds capable of inhibiting the VHL/HIF axis can potentially become therapeutic reagents for the treatment of metastatic PPGLs. For example, the HSP90 inhibitors, 17-N-allylamino-17-demethoxy geldanamycin (17-AAG) and 17-dimethylaminoethylamino-17-demethoxygeldanamycin (17-DMAG) (161–163), and histone deacetylase inhibitor, vorinostat (164, 165), are capable of inducing HIF-α degradation. Topotecan can downregulate HIF-α by inhibiting topoisomerase I (TOP-I) (166–168). Of note, topotecan has already been used as a therapeutic reagent for the treatment of metastatic ovarian carcinoma, recurrent small cell lung cancer, and recurrent cervical cancer (169–171). Acriflavine can inhibit dimerization between HIF-α and HIF-β and subsequently repress the expression of HIFs target genes (172). 2-Methoxyestradiol (2-ME), an active metabolite of 17β-estradiol, can inhibit the synthesis, nuclear translocation, and transcriptional activity of HIF-α (173, 174). In addition, an antiplatelet aggregation agent YC-1 can not only suppress HIFs transcriptional activity by inhibiting p300 recruitment but also promote HIF-α degradation by enhancing FIH binding (175). Finally, two anthracyclines, doxorubicin and daunorubicin, have been demonstrated to inhibit the expression of HIFs targets efficiently by interrupting HIF-α recruitment (176).

There are also compounds inhibiting HIF-1α synthesis. For example, PX-478 is capable of downregulating both the mRNA and protein levels of HIF-1α (177–179). EZN-2208 (PEG-SN38) can downregulate the expression of HIF-1α in lymphocytic leukemia (180). By hybridizing with HIF-1α mRNA, EZN-2968, a 3rd generation antisense oligonucleotide, can specifically inhibit HIF-1α translation (181, 182). Chetomin is capable of repressing xenograft growth *in vivo* by disrupting HIF-1α and p300 interaction (183). Finally, there is a myriad of compounds including echinomycin, CAY10585, KC7F2, glyceollins, bisphenol A, LW6, PX-12, cryptotanshinone (CPT), cyclo-CLLFVY, and indenopyrazoles 21 that have all been validated as selective inhibitors of HIF-1α with different molecular mechanisms (184–196).

## Conclusion

The VHL/HIF axis plays an important role in oxygen homeostasis and cellular metabolism in both physiology and pathology. Dysregulation of this axis due to either germline mutations, somatic mutations, and epigenetic dysregulation can be involved in tumorigenesis and progression of different cancer types including PPGLs. Mechanistically, by reprogramming metabolic pathways the abnormally activated HIFs drive cancer cells toward aerobic glycolysis. Based on the underlying molecular mechanisms of VHL/HIF axis in PPGLs development, a wide spectrum of drugs specifically targeting this axis have been and will continue to be developed as PPGL therapeutics. With a better understanding of the relationship between VHL/HIF axis and PPGLs, more accurate diagnosis and prognosis of PPGLs, as well as efficacious therapeutics against PPGLs, are expected in the near future.

## Author Contributions

SP, QL, JZ, XT, JX, and YH contributed to the writing of the manuscript. NS, JJ, and DZ provided consultation and contributed to the revising of the manuscript. All authors contributed to the article and approved the submitted version.

## Funding

This work was supported by the National Natural Science Foundation of China (81972398, JJ) and University Research Project of Army Medical University (218XLC3073, JZ).

## Conflict of Interest

The authors declare that the research was conducted in the absence of any commercial or financial relationships that could be construed as a potential conflict of interest.

## References

[1] Lenders JW Eisenhofer G Mannelli M Pacak K Phaeochromocytoma. Lancet (2005) 366(9486):665–75. 10.1016/S0140-6736(05)67139-5 16112304

[2] Stenstrom G Svardsudd K Pheochromocytoma in Sweden 1958-1981. An analysis of the National Cancer Registry Data. Acta Med Scand (1986) 220(3):225–32. 10.1111/j.0954-6820.1986.tb02755.x 3776697

[3] Ariton M Juan CS AvRuskin TW Pheochromocytoma: clinical observations from a Brooklyn tertiary hospital. Endocr Pract (2000) 6(3):249–52. 10.4158/EP.6.3.249 11421540

[4] Beard CM Sheps SG Kurland LT Carney JA Lie JT Occurrence of pheochromocytoma in Rochester, Minnesota, 1950 through 1979. Mayo Clin Proc (1983) 58(12):802–4. 6645626

[5] Eisenhofer G Pacak K Huynh TT Qin N Bratslavsky G Linehan WM Catecholamine metabolomic and secretory phenotypes in phaeochromocytoma. Endocr Relat Cancer (2011) 18(1):97–111. 10.1677/ERC-10-0211 21051559 PMC3671349

[6] Nomura K Kimura H Shimizu S Kodama H Okamoto T Obara T Survival of patients with metastatic malignant pheochromocytoma and efficacy of combined cyclophosphamide, vincristine, and dacarbazine chemotherapy. J Clin Endocrinol Metab (2009) 94(8):2850–6. 10.1210/jc.2008-2697 19470630

[7] Welander J Soderkvist P Gimm O Genetics and clinical characteristics of hereditary pheochromocytomas and paragangliomas. Endocr Relat Cancer (2011) 18(6):R253–76. 10.1530/ERC-11-0170 22041710

[8] Ayala-Ramirez M Feng L Johnson MM Ejaz S Habra MA Rich T Clinical risk factors for malignancy and overall survival in patients with pheochromocytomas and sympathetic paragangliomas: primary tumor size and primary tumor location as prognostic indicators. J Clin Endocrinol Metab (2011) 96(3):717–25. 10.1210/jc.2010-1946 21190975

[9] Taieb D Pacak K Genetic Determinants of Pheochromocytoma and Paraganglioma Imaging Phenotypes. J Nucl Med (2020) 61(5):643–5. 10.2967/jnumed.120.245613 PMC7198379 32198315

[10] Dahia PL Pheochromocytoma and paraganglioma pathogenesis: learning from genetic heterogeneity. Nat Rev Cancer (2014) 14(2):108–19. 10.1038/nrc3648 24442145

[11] Fishbein L Leshchiner I Walter V Danilova L Robertson AG Johnson AR Comprehensive Molecular Characterization of Pheochromocytoma and Paraganglioma. Cancer Cell (2017) 31(2):181–93. 10.1016/j.ccell.2017.01.001 PMC5643159 28162975

[12] Samanta D Prabhakar NR Semenza GL Systems biology of oxygen homeostasis. Wiley Interdiscip Rev Syst Biol Med (2017) 9(4). 10.1002/wsbm.1382 PMC5606335 28221004

[13] Wu D Rastinejad F Structural characterization of mammalian bHLH-PAS transcription factors. Curr Opin Struct Biol (2017) 43:1–9. 10.1016/j.sbi.2016.09.011 27721191 PMC5382129

[14] Yamashita K Discher DJ Hu J Bishopric NH Webster KA Molecular regulation of the endothelin-1 gene by hypoxia. Contributions of hypoxia-inducible factor-1, activator protein-1, GATA-2, AND p300/CBP. J Biol Chem (2001) 276(16):12645–53. 10.1074/jbc.M011344200 11278891

[15] Dayan F Roux D Brahimi-Horn MC Pouyssegur J Mazure NM The oxygen sensor factor-inhibiting hypoxia-inducible factor-1 controls expression of distinct genes through the bifunctional transcriptional character of hypoxia-inducible factor-1alpha. Cancer Res (2006) 66(7):3688–98. 10.1158/0008-5472.CAN-05-4564 16585195

[16] Kaelin WG Jr. Ratcliffe PJ Oxygen sensing by metazoans: the central role of the HIF hydroxylase pathway. Mol Cell (2008) 30(4):393–402. 10.1016/j.molcel.2008.04.009 18498744

[17] Bruick RK McKnight SL A conserved family of prolyl-4-hydroxylases that modify HIF. Science (2001) 294(5545):1337–40. 10.1126/science.1066373 11598268

[18] Favier J Buffet A Gimenez-Roqueplo AP HIF2A mutations in paraganglioma with polycythemia. N Engl J Med (2012) 367(22):2161. 10.1056/NEJMc1211953 23190243

[19] Ivan M Haberberger T Gervasi DC Michelson KS Gunzler V Kondo K Biochemical purification and pharmacological inhibition of a mammalian prolyl hydroxylase acting on hypoxia-inducible factor. Proc Natl Acad Sci USA (2002) 99(21):13459–64. 10.1073/pnas.192342099 PMC129695 12351678

[20] Baek JH Mahon PC Oh J Kelly B Krishnamachary B Pearson M OS-9 interacts with hypoxia-inducible factor 1alpha and prolyl hydroxylases to promote oxygen-dependent degradation of HIF-1alpha. Mol Cell (2005) 17(4):503–12. 10.1016/j.molcel.2005.01.011 15721254

[21] Keith B Johnson RS Simon MC HIF1alpha and HIF2alpha: sibling rivalry in hypoxic tumour growth and progression. Nat Rev Cancer (2011) 12(1):9–22. 10.1038/nrc3183 22169972 PMC3401912

[22] Al Tameemi W Dale TP Al-Jumaily RMK Forsyth NR Hypoxia-Modified Cancer Cell Metabolism. Front Cell Dev Biol (2019) 7:4. 10.3389/fcell.2019.00004 30761299 PMC6362613

[23] Bertout JA Patel SA Simon MC The impact of O2 availability on human cancer. Nat Rev Cancer (2008) 8(12):967–75. 10.1038/nrc2540 PMC3140692 18987634

[24] Triner D Shah YM Hypoxia-inducible factors: a central link between inflammation and cancer. J Clin Invest (2016) 126(10):3689–98. 10.1172/JCI84430 PMC5096825 27525434

[25] Hu CJ Wang LY Chodosh LA Keith B Simon MC Differential roles of hypoxia-inducible factor 1alpha (HIF-1alpha) and HIF-2alpha in hypoxic gene regulation. Mol Cell Biol (2003) 23(24):9361–74. 10.1128/mcb.23.24.9361-9374.2003 PMC309606 14645546

[26] Liu Q Wang Y Tong D Liu G Yuan W Zhang J A Somatic HIF2alpha Mutation-Induced Multiple and Recurrent Pheochromocytoma/Paraganglioma with Polycythemia: Clinical Study with Literature Review. Endocr Pathol (2017) 28(1):75–82. 10.1007/s12022-017-9469-4 28116635

[27] Heikkila M Pasanen A Kivirikko KI Myllyharju J Roles of the human hypoxia-inducible factor (HIF)-3alpha variants in the hypoxia response. Cell Mol Life Sci (2011) 68(23):3885–901. 10.1007/s00018-011-0679-5 PMC11114783 21479871

[28] Jaakkola P Mole DR Tian YM Wilson MI Gielbert J Gaskell SJ Targeting of HIF-alpha to the von Hippel-Lindau ubiquitylation complex by O2-regulated prolyl hydroxylation. Science (2001) 292(5516):468–72. 10.1126/science.1059796 11292861

[29] Loboda A Jozkowicz A Dulak J HIF-1 and HIF-2 transcription factors–similar but not identical. Mol Cells (2010) 29(5):435–42. 10.1007/s10059-010-0067-2 20396958

[30] Semenza GL Oxygen sensing, homeostasis, and disease. N Engl J Med (2011) 365(6):537–47. 10.1056/NEJMra1011165 21830968

[31] Jochmanova I Zhuang Z Pacak K Pheochromocytoma: Gasping for Air. Horm Cancer (2015) 6(5-6):191–205. 10.1007/s12672-015-0231-4 26138106 PMC10355934

[32] Kaelin WG Jr. The von Hippel-Lindau tumour suppressor protein: O2 sensing and cancer. Nat Rev Cancer (2008) 8(11):865–73. 10.1038/nrc2502 18923434

[33] Pouyssegur J Dayan F Mazure NM Hypoxia signalling in cancer and approaches to enforce tumour regression. Nature (2006) 441(7092):437–43. 10.1038/nature04871 16724055

[34] Gossage L Eisen T Maher ER VHL, the story of a tumour suppressor gene. Nat Rev Cancer (2015) 15(1):55–64. 10.1038/nrc3844 25533676

[35] Richard S Graff J Lindau J Resche F Von Hippel-Lindau disease. Lancet (2004) 363(9416):1231–4. 10.1016/S0140-6736(04)15957-6 15081659

[36] Lonser RR Glenn GM Walther M Chew EY Libutti SK Linehan WM von Hippel-Lindau disease. Lancet (2003) 361(9374):2059–67. 10.1016/S0140-6736(03)13643-4 12814730

[37] Chittiboina P Lonser RR Von Hippel-Lindau disease. Handb Clin Neurol (2015) 132:139–56. 10.1016/B978-0-444-62702-5.00010-X PMC5121930 26564077

[38] Richards FM Molecular pathology of von HippelLindau disease and the VHL tumour suppressor gene. Expert Rev Mol Med (2001) 2001:1–27. 10.1017/S1462399401002654 14987375

[39] Clifford SC Cockman ME Smallwood AC Mole DR Woodward ER Maxwell PH Contrasting effects on HIF-1alpha regulation by disease-causing pVHL mutations correlate with patterns of tumourigenesis in von Hippel-Lindau disease. Hum Mol Genet (2001) 10(10):1029–38. 10.1093/hmg/10.10.1029 11331613

[40] Zbar B Kishida T Chen F Schmidt L Maher ER Richards FM Germline mutations in the Von Hippel-Lindau disease (VHL) gene in families from North America, Europe, and Japan. Hum Mutat (1996) 8(4):348–57. 10.1002/(SICI)1098-1004(1996)8:4<348::AID-HUMU8>3.0.CO;2-3 8956040

[41] Liu Q Yuan G Tong D Liu G Yi Y Zhang J Novel genotype-phenotype correlations in five Chinese families with Von Hippel-Lindau disease. Endocr Connect (2018) 7(7):870–8. 10.1530/EC-18-0167 PMC6026882 29871882

[42] Nordstrom-O’Brien M van der Luijt RB van Rooijen E van den Ouweland AM Majoor-Krakauer DF Lolkema MP Genetic analysis of von Hippel-Lindau disease. Hum Mutat (2010) 31(5):521–37. 10.1002/humu.21219 20151405

[43] Beroud C Joly D Gallou C Staroz F Orfanelli MT Junien C Software and database for the analysis of mutations in the VHL gene. Nucleic Acids Res (1998) 26(1):256–8. 10.1093/nar/26.1.256 PMC147207 9399847

[44] Garcia A Matias-Guiu X Cabezas R Chico A Prat J Baiget M Molecular diagnosis of von Hippel-Lindau disease in a kindred with a predominance of familial phaeochromocytoma. Clin Endocrinol (Oxf) (1997) 46(3):359–63. 10.1046/j.1365-2265.1997.00149.x 9156047

[45] Ang SO Chen H Hirota K Gordeuk VR Jelinek J Guan Y Disruption of oxygen homeostasis underlies congenital Chuvash polycythemia. Nat Genet (2002) 32(4):614–21. 10.1038/ng1019 12415268

[46] Gallou C Chauveau D Richard S Joly D Giraud S Olschwang S Genotype-phenotype correlation in von Hippel-Lindau families with renal lesions. Hum Mutat (2004) 24(3):215–24. 10.1002/humu.20082 15300849

[47] Weirich G Klein B Wohl T Engelhardt D Brauch H VHL2C phenotype in a German von Hippel-Lindau family with concurrent VHL germline mutations P81S and L188V. J Clin Endocrinol Metab (2002) 87(11):5241–6. 10.1210/jc.2002-020651 12414898

[48] Fukino K Teramoto A Adachi K Takahashi H Emi M A family with hydrocephalus as a complication of cerebellar hemangioblastoma: identification of Pro157Leu mutation in the VHL gene. J Hum Genet (2000) 45(1):47–51. 10.1007/s100380050009 10697963

[49] Hong B Ma K Zhou J Zhang J Wang J Liu S Frequent Mutations of VHL Gene and the Clinical Phenotypes in the Largest Chinese Cohort With Von Hippel-Lindau Disease. Front Genet (2019) 10:867. 10.3389/fgene.2019.00867 31620170 PMC6759728

[50] Martin RL Walpole I Goldblatt J Identification of two sporadically derived mutations in the Von Hippel-Lindau gene. Hum Mutat (1996) 7(2):185. 10.1002/(SICI)1098-1004(1996)7:2<185::AID-HUMU22>3.0.CO;2-Y 8829648

[51] Crossey PA Foster K Richards FM Phipps ME Latif F Tory K Molecular genetic investigations of the mechanism of tumourigenesis in von Hippel-Lindau disease: analysis of allele loss in VHL tumours. Hum Genet (1994) 93(1):53–8. 10.1007/BF00218913 8270255

[52] Wang H Shepard MJ Zhang C Dong L Walker D Guedez L Deletion of the von Hippel-Lindau Gene in Hemangioblasts Causes Hemangioblastoma-like Lesions in Murine Retina. Cancer Res (2018) 78(5):1266–74. 10.1158/0008-5472.CAN-17-1718 PMC7446935 29301791

[53] Lee JS Lee JH Lee KE Kim JH Hong JM Ra EK Genotype-phenotype analysis of von Hippel-Lindau syndrome in Korean families: HIF-alpha binding site missense mutations elevate age-specific risk for CNS hemangioblastoma. BMC Med Genet (2016) 17(1):48. 10.1186/s12881-016-0306-2 27439424 PMC4955248

[54] Ong KR Woodward ER Killick P Lim C Macdonald F Maher ER Genotype-phenotype correlations in von Hippel-Lindau disease. Hum Mutat (2007) 28(2):143–9. 10.1002/humu.20385 17024664

[55] Liu SJ Wang JY Peng SH Li T Ning XH Hong BA Genotype and phenotype correlation in von Hippel-Lindau disease based on alteration of the HIF-alpha binding site in VHL protein. Genet Med (2018) 20(10):1266–73. 10.1038/gim.2017.261 29595810

[56] Bishop T Lau KW Epstein AC Kim SK Jiang M O’Rourke D Genetic analysis of pathways regulated by the von Hippel-Lindau tumor suppressor in Caenorhabditis elegans. PloS Biol (2004) 2(10):e289. 10.1371/journal.pbio.0020289 15361934 PMC515368

[57] Bommi-Reddy A Almeciga I Sawyer J Geisen C Li W Harlow E Kinase requirements in human cells: III. Altered kinase requirements in VHL-/- cancer cells detected in a pilot synthetic lethal screen. Proc Natl Acad Sci USA (2008) 105(43):16484–9. 10.1073/pnas.0806574105 PMC2575446 18948595

[58] Ohh M Yauch RL Lonergan KM Whaley JM Stemmer-Rachamimov AO Louis DN The von Hippel-Lindau tumor suppressor protein is required for proper assembly of an extracellular fibronectin matrix. Mol Cell (1998) 1(7):959–68. 10.1016/s1097-2765(00)80096-9 9651579

[59] Knudson AG Jr. Mutation and cancer: statistical study of retinoblastoma. Proc Natl Acad Sci U.S.A. (1971) 68(4):820–3. 10.1073/pnas.68.4.820 PMC389051 5279523

[60] Joosten SC Smits KM Aarts MJ Melotte V Koch A Tjan-Heijnen VC Epigenetics in renal cell cancer: mechanisms and clinical applications. Nat Rev Urol (2018) 15(7):430–51. 10.1038/s41585-018-0023-z 29867106

[61] Andreasson A Kiss NB Caramuta S Sulaiman L Svahn F Backdahl M The VHL gene is epigenetically inactivated in pheochromocytomas and abdominal paragangliomas. Epigenetics (2013) 8(12):1347–54. 10.4161/epi.26686 PMC3933494 24149047

[62] Morris MR Hughes DJ Tian YM Ricketts CJ Lau KW Gentle D Mutation analysis of hypoxia-inducible factors HIF1A and HIF2A in renal cell carcinoma. Anticancer Res (2009) 29(11):4337–43. 20032376

[63] Gladek I Ferdin J Horvat S Calin GA Kunej T HIF1A gene polymorphisms and human diseases: Graphical review of 97 association studies. Genes Chromosomes Cancer (2017) 56(6):439–52. 10.1002/gcc.22449 PMC5395341 28165644

[64] Percy MJ Beer PA Campbell G Dekker AW Green AR Oscier D Novel exon 12 mutations in the HIF2A gene associated with erythrocytosis. Blood (2008) 111(11):5400–2. 10.1182/blood-2008-02-137703 PMC2396730 18378852

[65] Percy MJ Furlow PW Lucas GS Li X Lappin TR McMullin MF A gain-of-function mutation in the HIF2A gene in familial erythrocytosis. N Engl J Med (2008) 358(2):162–8. 10.1056/NEJMoa073123 PMC2295209 18184961

[66] Liu Q Tong D Liu G Yi Y Zhang D Zhang J HIF2A germline-mutation-induced polycythemia in a patient with VHL-associated renal-cell carcinoma. Cancer Biol Ther (2017) 18(12):944–7. 10.1080/15384047.2017.1394553 PMC5718818 29172931

[67] Lorenzo FR Yang C Ng Tang Fui M Vankayalapati H Zhuang Z Huynh T A novel EPAS1/HIF2A germline mutation in a congenital polycythemia with paraganglioma. J Mol Med (Berl) (2013) 91(4):507–12. 10.1007/s00109-012-0967-z PMC3570726 23090011

[68] Comino-Mendez I de Cubas AA Bernal C Alvarez-Escola C Sanchez-Malo C Ramirez-Tortosa CL Tumoral EPAS1 (HIF2A) mutations explain sporadic pheochromocytoma and paraganglioma in the absence of erythrocytosis. Hum Mol Genet (2013) 22(11):2169–76. 10.1093/hmg/ddt069 23418310

[69] Zhuang Z Yang C Lorenzo F Merino M Fojo T Kebebew E Somatic HIF2A gain-of-function mutations in paraganglioma with polycythemia. N Engl J Med (2012) 367(10):922–30. 10.1056/NEJMoa1205119 PMC3432945 22931260

[70] Pacak K Jochmanova I Prodanov T Yang C Merino MJ Fojo T New syndrome of paraganglioma and somatostatinoma associated with polycythemia. J Clin Oncol (2013) 31(13):1690–8. 10.1200/JCO.2012.47.1912 PMC3807138 23509317

[71] Toledo RA Qin Y Srikantan S Morales NP Li Q Deng Y In vivo and in vitro oncogenic effects of HIF2A mutations in pheochromocytomas and paragangliomas. Endocr Relat Cancer (2013) 20(3):349–59. 10.1530/ERC-13-0101 PMC5944295 23533246

[72] Buffet A Smati S Mansuy L Menara M Lebras M Heymann MF Mosaicism in HIF2A-related polycythemia-paraganglioma syndrome. J Clin Endocrinol Metab (2014) 99(2):E369–73. 10.1210/jc.2013-2600 24276449

[73] Vaidya A Flores SK Cheng ZM Nicolas M Deng Y Opotowsky AR EPAS1 Mutations and Paragangliomas in Cyanotic Congenital Heart Disease. N Engl J Med (2018) 378(13):1259–61. 10.1056/NEJMc1716652 PMC5972530 29601261

[74] Minamishima YA Moslehi J Bardeesy N Cullen D Bronson RT Kaelin WG Jr. Somatic inactivation of the PHD2 prolyl hydroxylase causes polycythemia and congestive heart failure. Blood (2008) 111(6):3236–44. 10.1182/blood-2007-10-117812 PMC2265460 18096761

[75] Percy MJ Furlow PW Beer PA Lappin TR McMullin MF Lee FS A novel erythrocytosis-associated PHD2 mutation suggests the location of a HIF binding groove. Blood (2007) 110(6):2193–6. 10.1182/blood-2007-04-084434 PMC1976349 17579185

[76] Ladroue C Carcenac R Leporrier M Gad S Le Hello C Galateau-Salle F PHD2 mutation and congenital erythrocytosis with paraganglioma. N Engl J Med (2008) 359(25):2685–92. 10.1056/NEJMoa0806277 19092153

[77] Yang C Zhuang Z Fliedner SM Shankavaram U Sun MG Bullova P Germ-line PHD1 and PHD2 mutations detected in patients with pheochromocytoma/paraganglioma-polycythemia. J Mol Med (Berl) (2015) 93(1):93–104. 10.1007/s00109-014-1205-7 25263965

[78] Lee S Nakamura E Yang H Wei W Linggi MS Sajan MP Neuronal apoptosis linked to EglN3 prolyl hydroxylase and familial pheochromocytoma genes: developmental culling and cancer. Cancer Cell (2005) 8(2):155–67. 10.1016/j.ccr.2005.06.015 16098468

[79] Baysal BE Ferrell RE Willett-Brozick JE Lawrence EC Myssiorek D Bosch A Mutations in SDHD, a mitochondrial complex II gene, in hereditary paraganglioma. Science (2000) 287(5454):848–51. 10.1126/science.287.5454.848 10657297

[80] Niemann S Muller U Mutations in SDHC cause autosomal dominant paraganglioma, type 3. Nat Genet (2000) 26(3):268–70. 10.1038/81551 11062460

[81] Korpershoek E Favier J Gaal J Burnichon N van Gessel B Oudijk L SDHA immunohistochemistry detects germline SDHA gene mutations in apparently sporadic paragangliomas and pheochromocytomas. J Clin Endocrinol Metab (2011) 96(9):E1472–6. 10.1210/jc.2011-1043 21752896

[82] Burnichon N Briere JJ Libe R Vescovo L Riviere J Tissier F SDHA is a tumor suppressor gene causing paraganglioma. Hum Mol Genet (2010) 19(15):3011–20. 10.1093/hmg/ddq206 PMC2901140 20484225

[83] Selak MA Armour SM MacKenzie ED Boulahbel H Watson DG Mansfield KD Succinate links TCA cycle dysfunction to oncogenesis by inhibiting HIF-alpha prolyl hydroxylase. Cancer Cell (2005) 7(1):77–85. 10.1016/j.ccr.2004.11.022 15652751

[84] Philip B Ito K Moreno-Sanchez R Ralph SJ HIF expression and the role of hypoxic microenvironments within primary tumours as protective sites driving cancer stem cell renewal and metastatic progression. Carcinogenesis (2013) 34(8):1699–707. 10.1093/carcin/bgt209 23740838

[85] Pan Y Mansfield KD Bertozzi CC Rudenko V Chan DA Giaccia AJ Multiple factors affecting cellular redox status and energy metabolism modulate hypoxia-inducible factor prolyl hydroxylase activity in vivo and in vitro. Mol Cell Biol (2007) 27(3):912–25. 10.1128/MCB.01223-06 PMC1800695 17101781

[86] Wang L Lam G Thummel CS Med24 and Mdh2 are required for Drosophila larval salivary gland cell death. Dev Dyn (2010) 239(3):954–64. 10.1002/dvdy.22213 PMC2945606 20063412

[87] Calsina B Curras-Freixes M Buffet A Pons T Contreras L Leton R Role of MDH2 pathogenic variant in pheochromocytoma and paraganglioma patients. Genet Med (2018) 20(12):1652–62. 10.1038/s41436-018-0068-7 PMC7456538 30008476

[88] Castro-Vega LJ Buffet A De Cubas AA Cascon A Menara M Khalifa E Germline mutations in FH confer predisposition to malignant pheochromocytomas and paragangliomas. Hum Mol Genet (2014) 23(9):2440–6. 10.1093/hmg/ddt639 24334767

[89] Zhao S Lin Y Xu W Jiang W Zha Z Wang P Glioma-derived mutations in IDH1 dominantly inhibit IDH1 catalytic activity and induce HIF-1alpha. Science (2009) 324(5924):261–5. 10.1126/science.1170944 PMC3251015 19359588

[90] Baba M Hirai S Yamada-Okabe H Hamada K Tabuchi H Kobayashi K Loss of von Hippel-Lindau protein causes cell density dependent deregulation of CyclinD1 expression through hypoxia-inducible factor. Oncogene (2003) 22(18):2728–38. 10.1038/sj.onc.1206373 12743597

[91] Hudson CC Liu M Chiang GG Otterness DM Loomis DC Kaper F Regulation of hypoxia-inducible factor 1alpha expression and function by the mammalian target of rapamycin. Mol Cell Biol (2002) 22(20):7004–14. 10.1128/mcb.22.20.7004-7014.2002 PMC139825 12242281

[92] Mayerhofer M Valent P Sperr WR Griffin JD Sillaber C BCR/ABL induces expression of vascular endothelial growth factor and its transcriptional activator, hypoxia inducible factor-1alpha, through a pathway involving phosphoinositide 3-kinase and the mammalian target of rapamycin. Blood (2002) 100(10):3767–75. 10.1182/blood-2002-01-0109 12393646

[93] Jochmanova I Yang C Zhuang Z Pacak K Hypoxia-inducible factor signaling in pheochromocytoma: turning the rudder in the right direction. J Natl Cancer Inst (2013) 105(17):1270–83. 10.1093/jnci/djt201 PMC3888279 23940289

[94] Vicha A Musil Z Pacak K Genetics of pheochromocytoma and paraganglioma syndromes: new advances and future treatment options. Curr Opin Endocrinol Diabetes Obes (2013) 20(3):186–91. 10.1097/MED.0b013e32835fcc45 PMC4711348 23481210

[95] Semenza GL A compendium of proteins that interact with HIF-1alpha. Exp Cell Res (2017) 356(2):128–35. 10.1016/j.yexcr.2017.03.041 PMC5541399 28336293

[96] Ajith TA Current insights and future perspectives of hypoxia-inducible factor-targeted therapy in cancer. J Basic Clin Physiol Pharmacol (2018) 30(1):11–8. 10.1515/jbcpp-2017-0167 30260792

[97] Shen C Kaelin WG Jr. The VHL/HIF axis in clear cell renal carcinoma. Semin Cancer Biol (2013) 23(1):18–25. 10.1016/j.semcancer.2012.06.001 22705278 PMC3663044

[98] Jang M Kim SS Lee J Cancer cell metabolism: implications for therapeutic targets. Exp Mol Med (2013) 45:e45. 10.1038/emm.2013.85 24091747 PMC3809361

[99] Zhong H De Marzo AM Laughner E Lim M Hilton DA Zagzag D Overexpression of hypoxia-inducible factor 1alpha in common human cancers and their metastases. Cancer Res (1999) 59(22):5830–5. 10582706

[100] Maxwell PH Dachs GU Gleadle JM Nicholls LG Harris AL Stratford IJ Hypoxia-inducible factor-1 modulates gene expression in solid tumors and influences both angiogenesis and tumor growth. Proc Natl Acad Sci USA (1997) 94(15):8104–9. 10.1073/pnas.94.15.8104 PMC21564 9223322

[101] Liberti MV Locasale JW The Warburg Effect: How Does it Benefit Cancer Cells? Trends Biochem Sci (2016) 41(3):211–8. 10.1016/j.tibs.2015.12.001 PMC4783224 26778478

[102] Vander Heiden MG Cantley LC Thompson CB Understanding the Warburg effect: the metabolic requirements of cell proliferation. Science (2009) 324(5930):1029–33. 10.1126/science.1160809 PMC2849637 19460998

[103] Semenza GL Regulation of cancer cell metabolism by hypoxia-inducible factor 1. Semin Cancer Biol (2009) 19(1):12–6. 10.1016/j.semcancer.2008.11.009 19114105

[104] Maxwell PH Pugh CW Ratcliffe PJ Activation of the HIF pathway in cancer. Curr Opin Genet Dev (2001) 11(3):293–9. 10.1016/s0959-437x(00)00193-3 11377966

[105] Masoud GN Li W HIF-1alpha pathway: role, regulation and intervention for cancer therapy. Acta Pharm Sin B (2015) 5(5):378–89. 10.1016/j.apsb.2015.05.007 PMC4629436 26579469

[106] Gordan JD Thompson CB Simon MC HIF and c-Myc: sibling rivals for control of cancer cell metabolism and proliferation. Cancer Cell (2007) 12(2):108–13. 10.1016/j.ccr.2007.07.006 PMC3215289 17692803

[107] Icard P Lincet H A global view of the biochemical pathways involved in the regulation of the metabolism of cancer cells. Biochim Biophys Acta (2012) 1826(2):423–33. 10.1016/j.bbcan.2012.07.001 22841746

[108] Kruspig B Zhivotovsky B Gogvadze V Mitochondrial substrates in cancer: drivers or passengers? Mitochondrion (2014) 19 Pt A:8–19. 10.1016/j.mito.2014.08.007 25179741

[109] Weidemann A Johnson RS Biology of HIF-1alpha. Cell Death Differ (2008) 15(4):621–7. 10.1038/cdd.2008.12 18259201

[110] Erlic Z Beuschlein F Metabolic Alterations in Patients with Pheochromocytoma. Exp Clin Endocrinol Diabetes (2019) 127(2-03):129–36. 10.1055/a-0649-0960 30011405

[111] Richter S Gieldon L Pang Y Peitzsch M Huynh T Leton R Metabolome-guided genomics to identify pathogenic variants in isocitrate dehydrogenase, fumarate hydratase, and succinate dehydrogenase genes in pheochromocytoma and paraganglioma. Genet Med (2019) 21(3):705–17. 10.1038/s41436-018-0106-5 PMC6353556 30050099

[112] Gottlieb E Tomlinson IP Mitochondrial tumour suppressors: a genetic and biochemical update. Nat Rev Cancer (2005) 5(11):857–66. 10.1038/nrc1737 16327764

[113] Richter S Peitzsch M Rapizzi E Lenders JW Qin N de Cubas AA Krebs cycle metabolite profiling for identification and stratification of pheochromocytomas/paragangliomas due to succinate dehydrogenase deficiency. J Clin Endocrinol Metab (2014) 99(10):3903–11. 10.1210/jc.2014-2151 PMC4184070 25014000

[114] Dang L White DW Gross S Bennett BD Bittinger MA Driggers EM Cancer-associated IDH1 mutations produce 2-hydroxyglutarate. Nature (2009) 462(7274):739–44. 10.1038/nature08617 PMC2818760 19935646

[115] Xiao M Yang H Xu W Ma S Lin H Zhu H Inhibition of alpha-KG-dependent histone and DNA demethylases by fumarate and succinate that are accumulated in mutations of FH and SDH tumor suppressors. Genes Dev (2012) 26(12):1326–38. 10.1101/gad.191056.112 PMC3387660 22677546

[116] Peng X Gao H Xu R Wang H Mei J Liu C The interplay between HIF-1alpha and noncoding RNAs in cancer. J Exp Clin Cancer Res (2020) 39(1):27. 10.1186/s13046-020-1535-y 32014012 PMC6998277

[117] Huang X Zuo J Emerging roles of miR-210 and other non-coding RNAs in the hypoxic response. Acta Biochim Biophys Sin (Shanghai) (2014) 46(3):220–32. 10.1093/abbs/gmt141 24395300

[118] Li L Huang K You Y Fu X Hu L Song L Hypoxia-induced miR-210 in epithelial ovarian cancer enhances cancer cell viability via promoting proliferation and inhibiting apoptosis. Int J Oncol (2014) 44(6):2111–20. 10.3892/ijo.2014.2368 24715221

[119] Ying Q Liang L Guo W Zha R Tian Q Huang S Hypoxia-inducible microRNA-210 augments the metastatic potential of tumor cells by targeting vacuole membrane protein 1 in hepatocellular carcinoma. Hepatology (2011) 54(6):2064–75. 10.1002/hep.24614 22144109

[120] Yang W Sun T Cao J Liu F Tian Y Zhu W Downregulation of miR-210 expression inhibits proliferation, induces apoptosis and enhances radiosensitivity in hypoxic human hepatoma cells in vitro. Exp Cell Res (2012) 318(8):944–54. 10.1016/j.yexcr.2012.02.010 22387901

[121] Calsina B Castro-Vega LJ Torres-Perez R Inglada-Perez L Curras-Freixes M Roldan-Romero JM Integrative multi-omics analysis identifies a prognostic miRNA signature and a targetable miR-21-3p/TSC2/mTOR axis in metastatic pheochromocytoma/paraganglioma. Theranostics (2019) 9(17):4946–58. 10.7150/thno.35458 PMC6691382 31410193

[122] Flippot R Beinse G Boileve A Vibert J Malouf GG Long non-coding RNAs in genitourinary malignancies: a whole new world. Nat Rev Urol (2019) 16(8):484–504. 10.1038/s41585-019-0195-1 31110275

[123] Kim HC Lee JS Kim SH So HS Woo CY Lee JL Sunitinib treatment for metastatic renal cell carcinoma in patients with von hippel-lindau disease. Cancer Res Treat (2013) 45(4):349–53. 10.4143/crt.2013.45.4.349 PMC3893333 24454008

[124] Semenza GL Defining the role of hypoxia-inducible factor 1 in cancer biology and therapeutics. Oncogene (2010) 29(5):625–34. 10.1038/onc.2009.441 PMC2969168 19946328

[125] Melillo G Hypoxia-inducible factor 1 inhibitors. Methods Enzymol (2007) 435:385–402. 10.1016/S0076-6879(07)35020-9 17998065

[126] Melillo G Targeting hypoxia cell signaling for cancer therapy. Cancer Metastasis Rev (2007) 26(2):341–52. 10.1007/s10555-007-9059-x 17415529

[127] Xia Y Choi HK Lee K Recent advances in hypoxia-inducible factor (HIF)-1 inhibitors. Eur J Med Chem (2012) 49:24–40. 10.1016/j.ejmech.2012.01.033 22305612

[128] Wang Y Li Z Zhang H Jin H Sun L Dong H HIF-1alpha and HIF-2alpha correlate with migration and invasion in gastric cancer. Cancer Biol Ther (2010) 10(4):376–82. 10.4161/cbt.10.4.12441 20559021

[129] Joshi S Singh AR Zulcic M Durden DL. A macrophage-dominant PI3K isoform controls hypoxia-induced HIF1alpha and HIF2alpha stability and tumor growth, angiogenesis, and metastasis. Mol Cancer Res (2014) 12(10):1520–31. 10.1158/1541-7786.MCR-13-0682 25103499

[130] Philips GK Atkins MB New agents and new targets for renal cell carcinoma. Am Soc Clin Oncol Educ Book (2014) e222–7. 10.14694/EdBook_AM.2014.34.e222 24857106

[131] Roskoski R Jr. Properties of FDA-approved small molecule protein kinase inhibitors. Pharmacol Res (2019) 144:19–50. 10.1016/j.phrs.2019.03.006 30877063

[132] McCormack PL Pazopanib: a review of its use in the management of advanced renal cell carcinoma. Drugs (2014) 74(10):1111–25. 10.1007/s40265-014-0243-3 24935162

[133] Faivre S Demetri G Sargent W Raymond E Molecular basis for sunitinib efficacy and future clinical development. Nat Rev Drug Discovery (2007) 6(9):734–45. 10.1038/nrd2380 17690708

[134] Saito Y Tanaka Y Aita Y Ishii KA Ikeda T Isobe K Sunitinib induces apoptosis in pheochromocytoma tumor cells by inhibiting VEGFR2/Akt/mTOR/S6K1 pathways through modulation of Bcl-2 and BAD. Am J Physiol Endocrinol Metab (2012) 302(6):E615–25. 10.1152/ajpendo.00035.2011 21878661

[135] Aita Y Ishii KA Saito Y Ikeda T Kawakami Y Shimano H Sunitinib inhibits catecholamine synthesis and secretion in pheochromocytoma tumor cells by blocking VEGF receptor 2 via PLC-gamma-related pathways. Am J Physiol Endocrinol Metab (2012) 303(8):E1006–14. 10.1152/ajpendo.00156.2012 22912364

[136] Joshua AM Ezzat S Asa SL Evans A Broom R Freeman M Rationale and evidence for sunitinib in the treatment of malignant paraganglioma/pheochromocytoma. J Clin Endocrinol Metab (2009) 94(1):5–9. 10.1210/jc.2008-1836 19001511

[137] Jimenez C Cabanillas ME Santarpia L Jonasch E Kyle KL Lano EA Use of the tyrosine kinase inhibitor sunitinib in a patient with von Hippel-Lindau disease: targeting angiogenic factors in pheochromocytoma and other von Hippel-Lindau disease-related tumors. J Clin Endocrinol Metab (2009) 94(2):386–91. 10.1210/jc.2008-1972 19017755

[138] Ayala-Ramirez M Chougnet CN Habra MA Palmer JL Leboulleux S Cabanillas ME Treatment with sunitinib for patients with progressive metastatic pheochromocytomas and sympathetic paragangliomas. J Clin Endocrinol Metab (2012) 97(11):4040–50. 10.1210/jc.2012-2356 PMC3683800 22965939

[139] Motzer RJ Hutson TE Cella D Reeves J Hawkins R Guo J Pazopanib versus sunitinib in metastatic renal-cell carcinoma. N Engl J Med (2013) 369(8):722–31. 10.1056/NEJMoa1303989 23964934

[140] Rini BI Escudier B Tomczak P Kaprin A Szczylik C Hutson TE Comparative effectiveness of axitinib versus sorafenib in advanced renal cell carcinoma (AXIS): a randomised phase 3 trial. Lancet (2011) 378(9807):1931–9. 10.1016/S0140-6736(11)61613-9 22056247

[141] Yuan G Liu Q Tong D Liu G Yi Y Zhang J A retrospective case study of sunitinib treatment in three patients with Von Hippel-Lindau disease. Cancer Biol Ther (2018) 19(9):766–72. 10.1080/15384047.2018.1470732 PMC6154838 29947576

[142] O’Kane GM Ezzat S Joshua AM Bourdeau I Leibowitz-Amit R Olney HJ A phase 2 trial of sunitinib in patients with progressive paraganglioma or pheochromocytoma: the SNIPP trial. Br J Cancer (2019) 120(12):1113–9. 10.1038/s41416-019-0474-x PMC6738062 31105270

[143] Facchin C Perez-Liva M Garofalakis A Viel T Certain A Balvay D Concurrent imaging of vascularization and metabolism in a mouse model of paraganglioma under anti-angiogenic treatment. Theranostics (2020) 10(8):3518–32. 10.7150/thno.40687 PMC7069082 32206105

[144] Denorme M Yon L Roux C Gonzalez BJ Baudin E Anouar Y Both sunitinib and sorafenib are effective treatments for pheochromocytoma in a xenograft model. Cancer Lett (2014) 352(2):236–44. 10.1016/j.canlet.2014.07.005 25016061

[145] Wallace EM Rizzi JP Han G Wehn PM Cao Z Du X A Small-Molecule Antagonist of HIF2alpha Is Efficacious in Preclinical Models of Renal Cell Carcinoma. Cancer Res (2016) 76(18):5491–500. 10.1158/0008-5472.CAN-16-0473 27635045

[146] Gunaldi M Kara IO Duman BB Afsar CU Ergin M Avci A A new approach to the treatment of metastatic paraganglioma: sorafenib. Cancer Res Treat (2014) 46(4):411–4. 10.4143/crt.2013.093 PMC4206070 25036577

[147] Jimenez C Fazeli S Roman-Gonzalez A Antiangiogenic therapies for pheochromocytoma and paraganglioma. Endocr Relat Cancer (2020) 27(7):R239–R54. 10.1530/ERC-20-0043 32369773

[148] Pichun MEB Edgerly M Velarde M Bates SE Daerr R Adams K Phase II clinical trial of axitinib in metastatic pheochromocytomas and paraganlgiomas (P/PG): Preliminary results. J Clin Oncol (2015) 33:457–. 10.1200/jco.2015.33.7_suppl.457

[149] Jasim S Suman VJ Jimenez C Harris P Sideras K Burton JK Phase II trial of pazopanib in advanced/progressive malignant pheochromocytoma and paraganglioma. Endocrine (2017) 57(2):220–5. 10.1007/s12020-017-1359-5 28685225

[150] van Geel RM Beijnen JH Schellens JH Concise drug review: pazopanib and axitinib. Oncologist (2012) 17(8):1081–9. 10.1634/theoncologist.2012-0055 PMC3425526 22733795

[151] Gross-Goupil M Kwon TG Eto M Ye D Miyake H Seo SI Axitinib versus placebo as an adjuvant treatment of renal cell carcinoma: results from the phase III, randomized ATLAS trial. Ann Oncol (2018) 29(12):2371–8. 10.1093/annonc/mdy454 PMC6311952 30346481

[152] Erbel PJ Card PB Karakuzu O Bruick RK Gardner KH Structural basis for PAS domain heterodimerization in the basic helix–loop–helix-PAS transcription factor hypoxia-inducible factor. Proc Natl Acad Sci USA (2003) 100(26):15504–9. 10.1073/pnas.2533374100 PMC307597 14668441

[153] Cho H Du X Rizzi JP Liberzon E Chakraborty AA Gao W On-target efficacy of a HIF-2alpha antagonist in preclinical kidney cancer models. Nature (7627) 2016) 539:107–11. 10.1038/nature19795 PMC5499381 27595393

[154] Chen W Hill H Christie A Kim MS Holloman E Pavia-Jimenez A Targeting renal cell carcinoma with a HIF-2 antagonist. Nature (2016) 539(7627):112–7. 10.1038/nature19796 PMC5340502 27595394

[155] Courtney KD Infante JR Lam ET Figlin RA Rini BI Brugarolas J Phase I Dose-Escalation Trial of PT2385, a First-in-Class Hypoxia-Inducible Factor-2alpha Antagonist in Patients With Previously Treated Advanced Clear Cell Renal Cell Carcinoma. J Clin Oncol (2018) 36(9):867–74. 10.1200/JCO.2017.74.2627 PMC5946714 29257710

[156] Toledo RA New HIF2alpha inhibitors: potential implications as therapeutics for advanced pheochromocytomas and paragangliomas. Endocr Relat Cancer (2017) 24(9):C9–C19. 10.1530/ERC-16-0479 28667082

[157] Xu R Wang K Rizzi JP Huang H Grina JA Schlachter ST 3-[(1S,2S,3R)-2,3-Difluoro-1-hydroxy-7-methylsulfonylindan-4-yl]oxy-5-fluorobenzo nitrile (PT2977), a Hypoxia-Inducible Factor 2alpha (HIF-2alpha) Inhibitor for the Treatment of Clear Cell Renal Cell Carcinoma. J Med Chem (2019) 62(15):6876–93. 10.1021/acs.jmedchem.9b00719 31282155

[158] Choueiri TK Plimack ER Bauer TM Merchan JR Papadopoulos KP McDermott DF (2019). A First-in-Human Phase 1/2 Trial of the Oral HIF-2α Inhibitor PT2977 in Patients with Advanced RCC, in: Presented at the 14th European International Kidney Cancer Symposium, Dubrovnik, Croatia, March 29–30. 10.1093/annonc/mdz249.010

[159] Favier J Briere JJ Burnichon N Riviere J Vescovo L Benit P The Warburg effect is genetically determined in inherited pheochromocytomas. PloS One (2009) 4(9):e7094. 10.1371/journal.pone.0007094 19763184 PMC2738974

[160] Morin A Goncalves J Moog S Castro-Vega LJ Job S Buffet A TET-Mediated Hypermethylation Primes SDH-Deficient Cells for HIF2alpha-Driven Mesenchymal Transition. Cell Rep (2020) 30(13):4551–66.e7. 10.1016/j.celrep.2020.03.022 32234487

[161] Newman B Liu Y Lee HF Sun D Wang Y HSP90 inhibitor 17-AAG selectively eradicates lymphoma stem cells. Cancer Res (2012) 72(17):4551–61. 10.1158/0008-5472.CAN-11-3600 PMC3443561 22751135

[162] Bohonowych JE Peng S Gopal U Hance MW Wing SB Argraves KM Comparative analysis of novel and conventional Hsp90 inhibitors on HIF activity and angiogenic potential in clear cell renal cell carcinoma: implications for clinical evaluation. BMC Cancer (2011) 11:520. 10.1186/1471-2407-11-520 22172030 PMC3259130

[163] Ibrahim NO Hahn T Franke C Stiehl DP Wirthner R Wenger RH Induction of the hypoxia-inducible factor system by low levels of heat shock protein 90 inhibitors. Cancer Res (2005) 65(23):11094–100. 10.1158/0008-5472.CAN-05-1877 16322259

[164] Wigerup C Pahlman S Bexell D Therapeutic targeting of hypoxia and hypoxia-inducible factors in cancer. Pharmacol Ther (2016) 164:152–69. 10.1016/j.pharmthera.2016.04.009 27139518

[165] Hutt DM Roth DM Vignaud H Cullin C Bouchecareilh M The histone deacetylase inhibitor, Vorinostat, represses hypoxia inducible factor 1 alpha expression through translational inhibition. PloS One (2014) 9(8):e106224. 10.1371/journal.pone.0106224 25166596 PMC4148404

[166] Puppo M Battaglia F Ottaviano C Delfino S Ribatti D Varesio L Topotecan inhibits vascular endothelial growth factor production and angiogenic activity induced by hypoxia in human neuroblastoma by targeting hypoxia-inducible factor-1alpha and -2alpha. Mol Cancer Ther (2008) 7(7):1974–84. 10.1158/1535-7163.MCT-07-2059 18645007

[167] Rapisarda A Uranchimeg B Sordet O Pommier Y Shoemaker RH Melillo G Topoisomerase I-mediated inhibition of hypoxia-inducible factor 1: mechanism and therapeutic implications. Cancer Res (2004) 64(4):1475–82. 10.1158/0008-5472.can-03-3139 14983893

[168] Bertozzi D Marinello J Manzo SG Fornari F Gramantieri L Capranico G camptothecin, modulates HIF-1alpha activity by changing miR expression patterns in human cancer cells. Mol Cancer Ther (2014) 13(1):239–48. 10.1158/1535-7163.MCT-13-0729 24252850

[169] Lorusso D Pietragalla A Mainenti S Masciullo V Di Vagno G Scambia G Review role of topotecan in gynaecological cancers: current indications and perspectives. Crit Rev Oncol Hematol (2010) 74(3):163–74. 10.1016/j.critrevonc.2009.08.001 19766512

[170] Horita N Yamamoto M Sato T Tsukahara T Nagakura H Tashiro K Topotecan for Relapsed Small-cell Lung Cancer: Systematic Review and Meta-Analysis of 1347 Patients. Sci Rep (2015) 5:15437. 10.1038/srep15437 26486755 PMC4614251

[171] Musa F Blank S Muggia F A pharmacokinetic evaluation of topotecan as a cervical cancer therapy. Expert Opin Drug Metab Toxicol (2013) 9(2):215–24. 10.1517/17425255.2013.758249 23320990

[172] Lee K Zhang H Qian DZ Rey S Liu JO Semenza GL Acriflavine inhibits HIF-1 dimerization, tumor growth, and vascularization. Proc Natl Acad Sci USA (2009) 106(42):17910–5. 10.1073/pnas.0909353106 PMC2764905 19805192

[173] Ma L Li G Zhu H Dong X Zhao D Jiang X 2-Methoxyestradiol synergizes with sorafenib to suppress hepatocellular carcinoma by simultaneously dysregulating hypoxia-inducible factor-1 and -2. Cancer Lett (2014) 355(1):96–105. 10.1016/j.canlet.2014.09.011 25218350

[174] Zhou X Liu C Lu J Zhu L Li M 2-Methoxyestradiol inhibits hypoxia-induced scleroderma fibroblast collagen synthesis by phosphatidylinositol 3-kinase/Akt/mTOR signalling. Rheumatol (Oxford) (2018) 57(9):1675–84. 10.1093/rheumatology/key166 29905853

[175] Li SH Shin DH Chun YS Lee MK Kim MS Park JW A novel mode of action of YC-1 in HIF inhibition: stimulation of FIH-dependent p300 dissociation from HIF-1{alpha}. Mol Cancer Ther (2008) 7(12):3729–38. 10.1158/1535-7163.MCT-08-0074 19074848

[176] Lee K Qian DZ Rey S Wei H Liu JO Semenza GL Anthracycline chemotherapy inhibits HIF-1 transcriptional activity and tumor-induced mobilization of circulating angiogenic cells. Proc Natl Acad Sci USA (2009) 106(7):2353–8. 10.1073/pnas.0812801106 PMC2650160 19168635

[177] Pan X Lv Y Effects and Mechanism of Action of PX-478 in Oxygen-Induced Retinopathy in Mice. Ophthalmic Res (2020) 63(2):182–93. 10.1159/000504023 31955159

[178] Lee K Kim HM A novel approach to cancer therapy using PX-478 as a HIF-1alpha inhibitor. Arch Pharm Res (2011) 34(10):1583–5. 10.1007/s12272-011-1021-3 22076756

[179] Sun K Halberg N Khan M Magalang UJ Scherer PE Selective inhibition of hypoxia-inducible factor 1alpha ameliorates adipose tissue dysfunction. Mol Cell Biol (2013) 33(5):904–17. 10.1128/MCB.00951-12 PMC3623075 23249949

[180] Coltella N Valsecchi R Ponente M Ponzoni M Bernardi R Synergistic Leukemia Eradication by Combined Treatment with Retinoic Acid and HIF Inhibition by EZN-2208 (PEG-SN38) in Preclinical Models of PML-RARalpha and PLZF-RARalpha-Driven Leukemia. Clin Cancer Res (2015) 21(16):3685–94. 10.1158/1078-0432.CCR-14-3022 25931453

[181] Jeong W Rapisarda A Park SR Kinders RJ Chen A Melillo G Pilot trial of EZN-2968, an antisense oligonucleotide inhibitor of hypoxia-inducible factor-1 alpha (HIF-1alpha), in patients with refractory solid tumors. Cancer Chemother Pharmacol (2014) 73(2):343–8. 10.1007/s00280-013-2362-z PMC8375568 24292632

[182] Greenberger LM Horak ID Filpula D Sapra P Westergaard M Frydenlund HF A RNA antagonist of hypoxia-inducible factor-1alpha, EZN-2968, inhibits tumor cell growth. Mol Cancer Ther (2008) 7(11):3598–608. 10.1158/1535-7163.MCT-08-0510 18974394

[183] Kung AL Zabludoff SD France DS Freedman SJ Tanner EA Vieira A Small molecule blockade of transcriptional coactivation of the hypoxia-inducible factor pathway. Cancer Cell (2004) 6(1):33–43. 10.1016/j.ccr.2004.06.009 15261140

[184] Kong D Park EJ Stephen AG Calvani M Cardellina JH Monks A Echinomycin, a small-molecule inhibitor of hypoxia-inducible factor-1 DNA-binding activity. Cancer Res (2005) 65(19):9047–55. 10.1158/0008-5472.CAN-05-1235 16204079

[185] Hu N Jiang D Huang E Liu X Li R Liang X BMP9-regulated angiogenic signaling plays an important role in the osteogenic differentiation of mesenchymal progenitor cells. J Cell Sci (2013) 126(Pt 2):532–41. 10.1242/jcs.114231 PMC3613181 23203800

[186] Narita T Yin S Gelin CF Moreno CS Yepes M Nicolaou KC Identification of a novel small molecule HIF-1alpha translation inhibitor. Clin Cancer Res (2009) 15(19):6128–36. 10.1158/1078-0432.CCR-08-3180 PMC2770235 19789328

[187] Lee SH Jee JG Bae JS Liu KH Lee YM A group of novel HIF-1alpha inhibitors, glyceollins, blocks HIF-1alpha synthesis and decreases its stability via inhibition of the PI3K/AKT/mTOR pathway and Hsp90 binding. J Cell Physiol (2015) 230(4):853–62. 10.1002/jcp.24813 25204544

[188] Pham TH Lecomte S Efstathiou T Ferriere F Pakdel F An Update on the Effects of Glyceollins on Human Health: Possible Anticancer Effects and Underlying Mechanisms. Nutrients (2019) 11(1):79. 10.3390/nu11010079 PMC6357109 30609801

[189] Kubo T Maezawa N Osada M Katsumura S Funae Y Imaoka S an environmental endocrine-disrupting chemical, inhibits hypoxic response via degradation of hypoxia-inducible factor 1alpha (HIF-1alpha): structural requirement of bisphenol A for degradation of HIF-1alpha. Biochem Biophys Res Commun (2004) 318(4):1006–11. 10.1016/j.bbrc.2004.04.125 15147973

[190] Fu B Xue J Li Z Shi X Jiang BH Fang J Chrysin inhibits expression of hypoxia-inducible factor-1alpha through reducing hypoxia-inducible factor-1alpha stability and inhibiting its protein synthesis. Mol Cancer Ther (2007) 6(1):220–6. 10.1158/1535-7163.MCT-06-0526 17237281

[191] Lee K Kang JE Park SK Jin Y Chung KS Kim HM LW6, a novel HIF-1 inhibitor, promotes proteasomal degradation of HIF-1alpha via upregulation of VHL in a colon cancer cell line. Biochem Pharmacol (2010) 80(7):982–9. 10.1016/j.bcp.2010.06.018 20599784

[192] Kim YH Coon A Baker AF Powis G Antitumor agent PX-12 inhibits HIF-1alpha protein levels through an Nrf2/PMF-1-mediated increase in spermidine/spermine acetyl transferase. Cancer Chemother Pharmacol (2011) 68(2):405–13. 10.1007/s00280-010-1500-0 PMC3107346 21069338

[193] Jordan BF Runquist M Raghunand N Gillies RJ Tate WR Powis G The thioredoxin-1 inhibitor 1-methylpropyl 2-imidazolyl disulfide (PX-12) decreases vascular permeability in tumor xenografts monitored by dynamic contrast enhanced magnetic resonance imaging. Clin Cancer Res (2005) 11(2 Pt 1):529–36. 15701837

[194] Zhang L Chen C Duanmu J Wu Y Tao J Yang A Cryptotanshinone inhibits the growth and invasion of colon cancer by suppressing inflammation and tumor angiogenesis through modulating MMP/TIMP system, PI3K/Akt/mTOR signaling and HIF-1alpha nuclear translocation. Int Immunopharmacol (2018) 65:429–37. 10.1016/j.intimp.2018.10.035 30388517

[195] Miranda E Nordgren IK Male AL Lawrence CE Hoakwie F Cuda F A cyclic peptide inhibitor of HIF-1 heterodimerization that inhibits hypoxia signaling in cancer cells. J Am Chem Soc (2013) 135(28):10418–25. 10.1021/ja402993u PMC3715890 23796364

[196] Minegishi H Fukashiro S Ban HS Nakamura H Discovery of Indenopyrazoles as a New Class of Hypoxia Inducible Factor (HIF)-1 Inhibitors. ACS Med Chem Lett (2013) 4(2):297–301. 10.1021/ml3004632 24900662 PMC4027554

